# A thumb-domain insertion balances processivity and fidelity in DNA polymerase ε

**DOI:** 10.1093/nar/gkag282

**Published:** 2026-03-31

**Authors:** Noopur Singh, Göran O Bylund, Erik Johansson

**Affiliations:** Department of Medical Biochemistry and Biophysics, Umeå University, Umeå 901 87, Sweden; Department of Medical Biochemistry and Biophysics, Umeå University, Umeå 901 87, Sweden; Department of Medical Biochemistry and Biophysics, Umeå University, Umeå 901 87, Sweden

## Abstract

Recent cryo-EM structures of human DNA polymerase ε (Pol ε) bound to PCNA position a Pol ε-specific thumb insertion (polymerase thumb insertion; PTI) adjacent to a PCNA protomer, suggesting a regulatory role in DNA synthesis. To define the functional contribution of this region, we generated alanine-substitution variants in the yeast Pol ε thumb domain, targeting the PTI (SLED_1131–1134_→AAAA; polε-SLED) and an adjacent conserved loop (PVTE_1101–1104_→AAAA; polε-PVTE and KPFN_1096–1099_→AAAA; polε-KPFN). polε-SLED displayed increased intrinsic processivity, efficient bypass of DNA secondary structures, and enhanced synthesis on long templates, consistent with reduced pausing. In contrast, a previous study extended this substitution to six amino acids, SLEDLD_1131-1136_→AAAAAA, and found a reversed effect, a reduced processivity, indicating that subtle perturbations in this insertion can have opposing functional consequences. polε-PVTE shifted polymerase activity toward exonuclease proofreading and was not fully rescued by PCNA on long templates, whereas polε-KPFN retained near–wild-type activity but showed increased sensitivity to secondary structures that was alleviated by PCNA. *In vivo*, the corresponding pol2-SLED allele caused a modest mutator phenotype, while pol2-PVTE and pol2-KPFN showed little or no increase. Together, these results indicate that the PTI fine-tunes intrinsic processivity and proofreading to maintain replication fidelity during leading-strand synthesis.

## Graphical Abstract

**Graphical Abstract:**
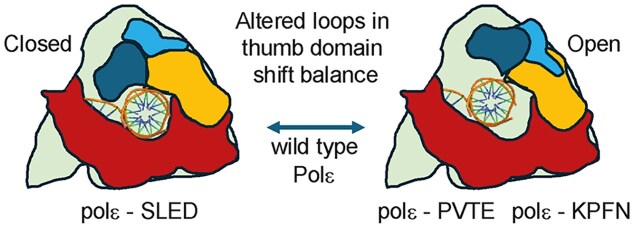

## Introduction

The catalytic core of B-family replicative polymerases adopts a classical right-handed arrangement, composed of palm, thumb, fingers, exonuclease, and N-terminal domain [1, 2]. In spite of this similarity in the overall arrangement of domains, a set of unique insertions makes the catalytic core of Pol ε different from other replicative polymerases. Such unique insertions are often hallmarks of evolution of new or altered functions within classically conserved protein folds [3, 4]. One of these insertions in Pol ε is the P-domain that enhances the processivity of Pol ε, independent of its interaction with PCNA [5, 6]. Another domain is located at the base of the thumb domain, was initially identified as important for the fidelity of Pol ε [7] and later named POPS, shown to influence the switching between the polymerase and exonuclease sites during proofreading [8]. A combination of other insertions coordinates the FeS cluster that is essential for the polymerase activity of Pol ε [9].

**Figure 1.**
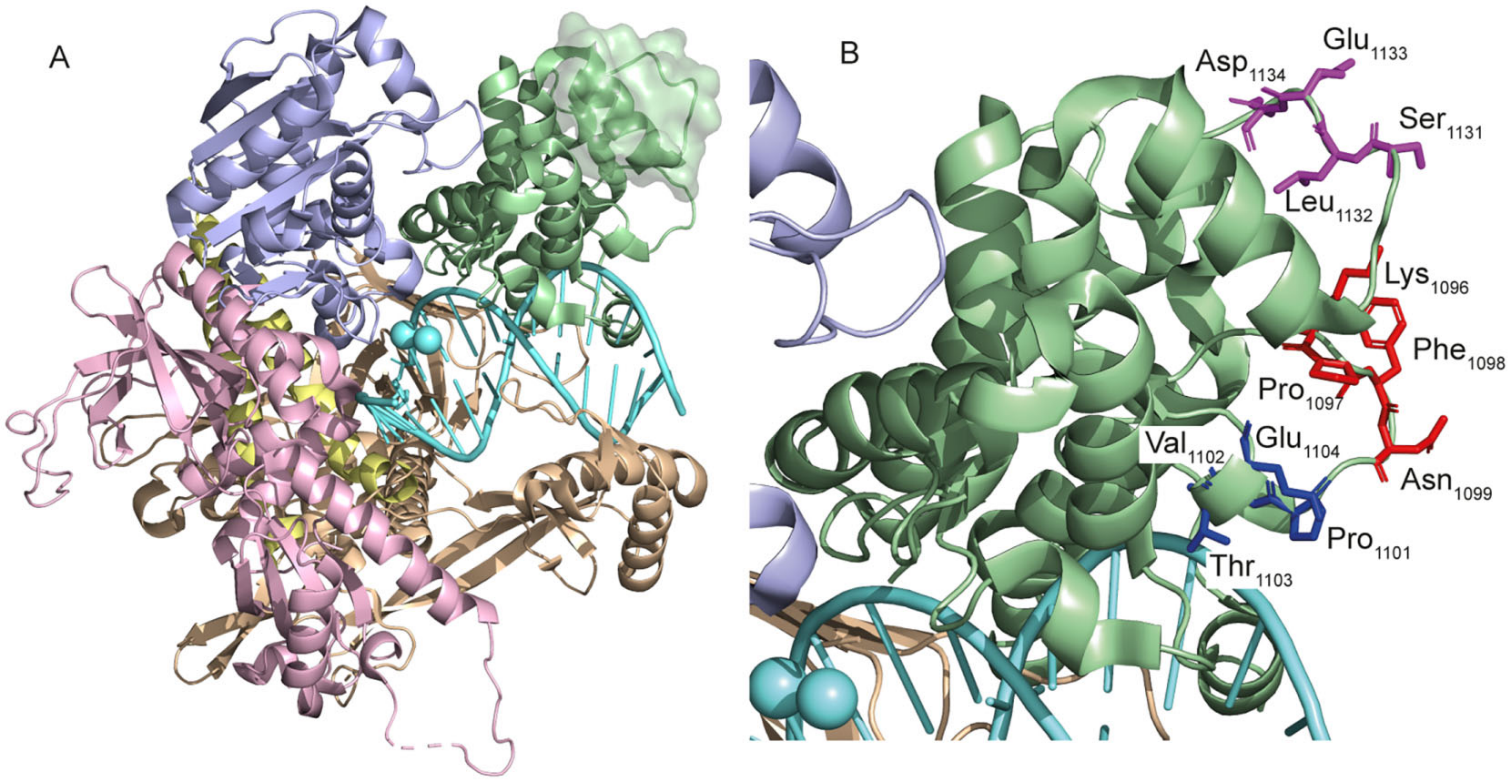
(**A**) Catalytic core region of Pol ε (PDB ID 4m8o [5]) showing the domain organization: palm and P-domain (wheat), thumb domain (green), fingers domain (yellow), exonuclease domain (blue), and N-terminal domain (pink). The polymerase-thumb-insertion (PTI) is shown with a green surface representation. (**B**) Side chains of the residues selected for amino acid substitutions _1131_SLED_1134_ (magenta) in PTI and _1096_KPFN_1099_ (red), _1101_PVTE_1104_ (blue) in a conserved thumb-domain loop.

An understudied insertion, comprising residues 1116–1140 in the thumb domain of yeast Pol ε, is also referred to as PTI (polymerase thumb domain insertion) (Fig. 1 and Supplementary Fig. S1) [5, 10]. It is well established that the thumb domain in all DNA polymerases stabilizes the interaction with the template by contacting the nascent duplex DNA [11]. A deletion or alanine substitution of six amino acids in the PTI was recently shown to disrupt the high processivity of Pol ε [10]. Another study showed that residue R988 in the thumb domain in yeast Pol ε is required for processive switching between the exonuclease and polymerase sites [12]. This observation was further supported by a more recent structural study with human Pol ε, where it was shown that the thumb domain together with the P-domain assists the transfer of mismatched DNA from the polymerase to exonuclease site, implicating a role during proofreading [13]. Also in earlier reports it was shown that the thumb domain participates in the switch between polymerizing and editing modes in family B polymerases lacking a P-domain, such as RB69 DNA polymerase and ϕ29 DNA polymerase [14, 15]. In addition, cryo-EM structures of human Pol ε–PCNA complex revealed that the thumb domain of human Pol ε may interact weakly with a protomer of PCNA. This interaction involves residues from a loop in the unique thumb domain insertion region (aa1102–1122 in human) and another conserved loop in the thumb domain (Supplementary Fig. S1) [13, 16]. However, it seems counterintuitive that Pol ε would have evolved an altogether new domain, the P-domain, to reduce the dependence on PCNA for processive DNA synthesis and simultaneously developed an insertion in the thumb domain to facilitate the interaction with PCNA, unless there is a specific reason for this.

To test how the PTI and neighboring thumb-domain loops contribute to Pol ε function, we engineered minimal alanine substitutions in yeast Pol ε: a PTI variant (SLED_1131–1134_→AAAA; polε-SLED) and two variants in adjacent conserved loops (PVTE_1101–1104_→AAAA; polε-PVTE and KPFN_1096–1099_→AAAA; polε-KPFN) (Fig. 1). Using primer-extension, holoenzyme, and single-hit processivity assays, together with tetrad analysis revealing potential growth defects and measurement of spontaneous mutation rates, we show that a PTI perturbation increases intrinsic processivity and enhances bypass of secondary structures but modestly elevates spontaneous mutagenesis, whereas substitutions in structurally conserved loops primarily promote pausing and/or exonucleolytic degradation. These findings support a model in which the PTI acts as a regulatory element that helps balance processivity and fidelity during leading-strand replication.

## Materials and methods

### Expression and purification of Pol ε and its variants

Point mutations were introduced into the pJL1 plasmid for overexpression of Pol2 with selected alanine substitutions using polymerase chain reaction (PCR)-based site-directed mutagenesis. The used primer pairs are listed in Supplementary Table S1. Wild-type Pol ε and alanine-substitution variants of Pol ε, namely polε-SLED, polε-PVTE, and polε-KPFN, were overexpressed in the *Saccharomyces cerevisiae* strain PY116 from two plasmids: pJL1, encoding Pol2, and pJL6, encoding Dpb2, Dpb3, and Dpb4. Expression of both plasmids was driven by the GAL1–10 promoter. Protein expression and purification were performed exactly as described previously [9]. RFC, PCNA, and RPA were purified as described in [17–19].

###  Primer-extension assays

#### Normal and hairpin-containing templates

Primer-extension reactions were carried out in RQ buffer containing 40 mM Tris–HCl (pH 7.8), 100 µg/ml bovine serum albumin (BSA), and 1 mM dithiothreitol (DTT). In the final reaction after mixing A and B (see below), the contribution of salt from proteins and DNA substrate rendered a final salt concentration of 65 mM NaAc. Reaction mixture A (10 µl) contained 25 nM DNA substrate (50-mer primer annealed to an 80-mer normal template or an 83-mer hairpin-containing template), 8 mM magnesium acetate (MgAc₂), and a 2× physiological dNTP mix (44 µM dATP, 22 µM dGTP, 78 µM dCTP, and 132 µM dTTP) in RQ buffer. Reaction mixture B (10 µl) contained 6 nM polymerase and 8 mM MgAc₂ in RQ buffer. Mixtures A and B were combined and incubated at 30°C for 0, 2, 5, or 15 min. Reactions were terminated by addition of 20 µl stop solution [95% formamide, 20 mM ethylenediaminetetraacetic acid (EDTA), and 0.1% bromophenol blue]. Samples were diluted four-fold in 0.5× stop solution, heated at 90°C for 15 min, and resolved on 10% denaturing polyacrylamide gels in 1× TBE buffer. Gels were scanned using an Amersham Typhoon scanner at 535 nm to detect the 5′-TET–labeled primer.

### Holoenzyme assays

Holoenzyme reactions (15 µl) contained 40 mM Tris–HCl (pH 7.8), 200 µg/ml BSA, 1 mM DTT, 8 mM MgAc₂, 125 mM sodium acetate (NaAc), 0.5 mM ATP, 100 µM dATP, dGTP, and dTTP, 50 µM dCTP, α-³²P-dCTP, 75 fmol single-primed pBluescript II SK(+), 10.5 pmol RPA, 1.15 pmol PCNA, 80 fmol RFC, and 143 fmol Pol ε or variant. Reactions were incubated at 30°C and stopped at the indicated time points by addition of 1 µl 0.5 M EDTA. Control reactions lacking RFC were incubated for a total of 8 min. Reaction products were purified using Cytiva G-50 columns, mixed with loading dye (10% sucrose, 0.1% bromophenol blue), and resolved on 1% alkaline agarose gels containing 30 mM NaOH and 2 mM EDTA. Gels were run at 30 V for 16 h, fixed in 5% trichloroacetic acid for 1 h, dried at 55°C, and analyzed by phosphorimaging using an Amersham Typhoon scanner.

### Processivity assays

The substrate for processivity assays was prepared by annealing M13mp18 single-stranded DNA (NEB) with a 5′-TET–labeled 35-mer oligonucleotide (TET-CCCAGTCACGACGTTGTAAAACGACGGCCAGTGCC) in equimolar amounts in 125 mM NaAc (pH 7.8). The mixture was heated to 70°C for 5 min and slowly cooled to room temperature.

Reactions were performed essentially as described for the holoenzyme assay, with the following modifications: (i) the dNTP mix contained 100 µM each of dATP, dGTP, dTTP, and dCTP; and (ii) reactions were conducted under single-hit conditions using a 40-fold molar excess of DNA substrate over polymerase. Reactions (15 µl) were stopped by addition of 8 µl stop solution and heated at 90°C for 15 min. A total of 7 µl of each reaction was resolved on 8% denaturing polyacrylamide gels in 1× TBE buffer and visualized using an Amersham Typhoon scanner.

### *In vivo* analysis

Yeast E134 strains (*ade5-1 lys2::insEA14 trp1-289 his7-2 leu2-3 112 ura3-52*) carrying pol2-SLED (_1131_SLED_1134_→AAAA), pol2-PVTE (_1101_PVTE_1104_→AAAA), or pol2-KPFN (_1096_KPFN_1099_→AAAA) alleles were constructed as described previously [9]. Mutations were identified by PCR screening and confirmed by DNA sequencing of heterozygous diploids.

Cellular fitness was assessed by tetrad dissection as described previously [20]. Temperature sensitivity was evaluated using spot dilution assays. Briefly, strains were grown on YPD plates at 30°C for 2 days. Single colonies were inoculated into 50 ml YPD medium and grown overnight at 30°C with shaking. Cultures were harvested, resuspended in water to an OD_600_ of 1.0, and subjected to fivefold serial dilutions. Aliquots (5 µl) were spotted onto YP agar plates, allowed to dry, and incubated at 30°C or 37°C for 48 h.

Spontaneous mutation rates were determined using fluctuation assays performed with 10 independent cultures per strain in two biological replicates (20 cultures total). Mutation rates were calculated as described previously [21, 22].

### Figures

Structural figures were generated using PyMOL (Schrödinger, LLC) and prepared for publication in Adobe Illustrator.

## Results

### Rationale for generation of mutants

Pol ε has a unique thumb domain insertion, residues 1116–1140 in Pol2 in yeast, that is not found in other B-family polymerases (Supplementary Fig. S2). The insertion is overall structurally conserved, but is less conserved when comparing the primary sequence from different eukaryotes. Despite that, a comparison between yeast and human Pol ε revealed only one amino acid difference, yeast SLED_1131–1134_ versus human SLQD_1118–1121_ (Supplementary Fig. S3A). To explore whether this unique thumb domain insertion could influence the interaction with PCNA, a SLED_1131–1134_→AAAA variant (from here on called Polε-SLED) was created in a loop region instead of well-defined secondary structural elements to keep structural distortions to a minimum (Fig. 1B). To be noted, a previously published six-amino acid deletion (SLEDLD_1131–1136_ deletion, from here on called pol2-6Δ) or alanine substitutions (SLEDLD_1131–1136_→AAAAAA, from here on called pol2-6A) overlapping with these amino acids extended into a short α-helix [10]. This may be important when comparing the properties of the two different Pol ε variants, as there are opposing functional consequences. Additionally, we generated two other Pol ε variants by substituting PVTE_1101–1104_→AAAA and KPFN_1096–1099_→AAAA (from here on called polε-PVTE and polε-KPFN, respectively) in a structurally conserved thumb domain region that faces PCNA and is also found in other B-family polymerases (Fig. 1 and Supplementary Fig. S4). The residues in polε-PVTE are conserved between human and yeast Polε, whereas polε-KPFN is not (Supplementary Fig. S3B and C). Although this region is structurally conserved among other B-family polymerases, the residue assignments to this region vary when aligning different B-family polymerases. It remained of interest to explore whether these residues contribute to the interaction with PCNA, given that they are oriented toward PCNA (Supplementary Fig. S4) and that interactions between DNA polymerases and PCNA are species-specific. Modification of two distinct regions of the thumb domain allowed functional differences to be isolated and attributed to specific regional alterations.

### Polymerase activity of polε-SLED, polε-PVTE, and Polε-KPFN variants

**Figure 2.**
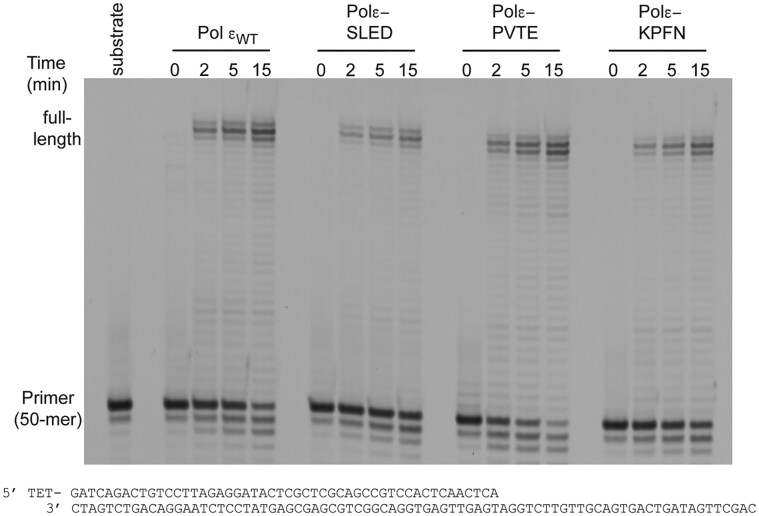
Denaturing PAGE (10%) showing the results of primer-extension assay with wild-type Pol ε and its variants. Extension of a 5′-TET-labeled 50-mer primer annealed to an 80-mer template. The reactions were stopped at 0, 2, 5, and 15 min, respectively.

**Figure 3.**
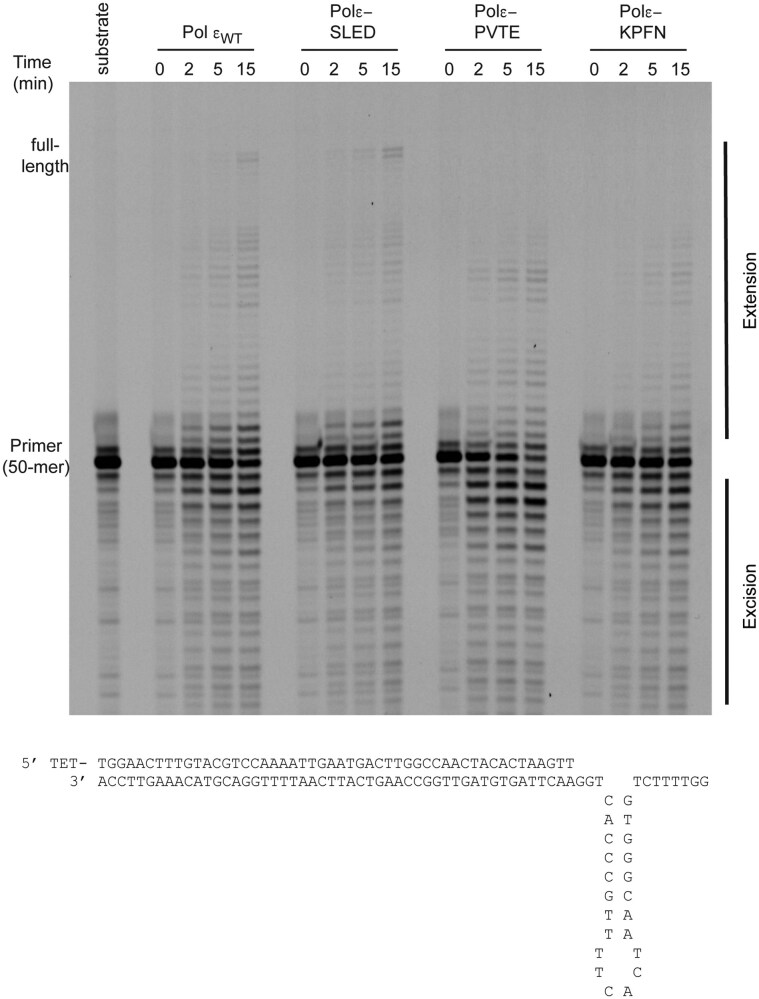
Denaturing PAGE (10%) showing the results of primer-extension assay with wild-type Pol ε and its variants. Extension of a 5′-TET-labeled 50-mer primer annealed to an 83-mer template containing a hairpin. The reactions were stopped at 0, 2, 5, and 15 min, respectively.

To explore whether the alanine substitutions would have a strong general negative impact on the polymerase activity, primer extension assays were carried out with a 5′-TET-labeled 50-mer primer annealed to an 80-mer template. All three variants, polε-SLED, polε-PVTE, and polε-KPFN, were catalytically active, extending a primer annealed to a single-stranded template to the end of the template (Fig. 2). After 15 min, wild-type Pol ε extended 50% of the primer to full-length products. The yield of polε-SLED, polε-PVTE, and polε-KPFN were 27%, 49%, and 39% full-length products, respectively (Fig. 2). However, when challenged by a hairpin on the template strand, wild-type Polε and the polε-SLED variant were able to extend 3% and 5% of the primer to the end of template, respectively (Fig. 3). It should be noted that even though the polε-SLED variant was less active than wild type Pol ε on the normal template, it was more competent than wild-type Pol ε when encountering a hairpin structure. Compare the yield of full-length products in Fig. 2 (wild-type Pol ε, 50%, and polε-SLED, 27%) with the yield of full-length products in Fig. 3 (wild-type Pol ε, 3%, and polε-SLED, 5%).

In contrast, polε-PVTE and polε-KPFN were inhibited by the hairpin structure and failed to reach the end of the template even after 15 min of incubation. When comparing degradation products from the inherent exonuclease activity in Pol ε, the polε-SLED variant produced less degradation products compared to wild-type Pol ε, the polε-KPFN variant was comparable to wild-type Pol ε, and the polε-PVTE variant showed a greater tendency to degrade the primer strand on both a normal and a hairpin-containing template. These primer extension assays revealed that the three different variants in the thumb domain were affected in different ways. The polε-SLED variant was potentially more efficient in bypassing the hairpin structure, the polε-KPFN variant stalled at the hairpin, and the polε-PVTE variant stalled at the hairpin and began degrading the primer. The underlying cause for these observations could be altered intrinsic processivity of Pol ε or balance between polymerase and exonuclease activity or a combination of both.

### PCNA-dependent DNA synthesis

Given that the mutant enzymes displayed polymerase activities comparable to wild-type Pol ε (50%–100%) on an unperturbed template but showed variable activities when challenged with a hairpin structure, it was investigated whether PCNA could compensate for these defects by enhancing processivity and stabilizing Pol ε on the template. Accordingly, all three mutants were examined in assays using a long single-stranded DNA template to test whether PCNA could suppress the observed defects.

**Figure 4.**
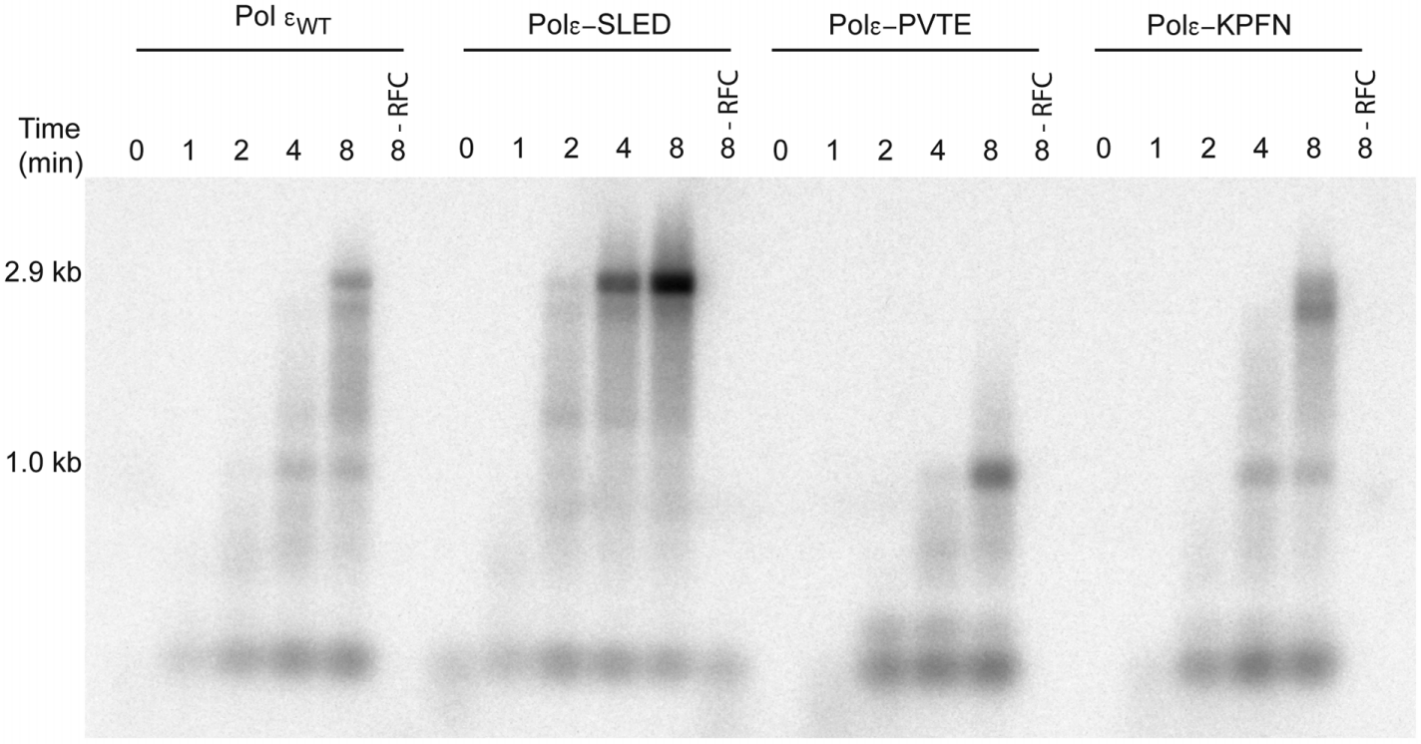
Alkaline agarose gel (1%) showing the impact of PCNA on leading strand synthesis by wild-type Pol ε and its variants in the holoenzyme assay. A single-primed pBluescript II SK (+) template (75 fmol) was replicated by four subunit Pol ε or its variant (143 fmol) in the presence of RPA (10.5 pmol), PCNA (1.15 pmol), and RFC (80 fmol). Individual reactions were stopped at 0, 4, and 8 min, respectively. Lanes labeled −RFC represents reactions performed in the absence of RFC.

A single-primed pBluescript II SK(+) template was used in these assays for two reasons: (i) defects in processive leading-strand synthesis become more apparent when polymerases are required to synthesize products over several kilobases, as compared with the relatively short templates used in primer-extension assays; and (ii) longer templates are more prone to forming sequence-specific secondary structures that can act as polymerase stall sites. Using this system, a time-dependent increase in product length was observed for wild-type Pol ε, with appearance of the full-length product (2.9 kb) after 8 min (Fig. 4). At 4 and 8 min, bands of intermediate length were also detected, likely representing products generated following intermittent stalling during synthesis.

Strikingly, the polε-SLED variant generated the full-length 2.9 kb product as early as at 2 min, with further increases in product accumulation at 4 and 8 min (Fig. 4). Compared with wild-type Pol ε, the increased yield of full-length products for polε-SLED correlated with a marked reduction, or near absence, of intermediate species. These results indicate that the polε-SLED variant is more proficient at bypassing stall sites associated with secondary structure formation, consistent with our observations from hairpin-containing primer-extension assays.

In contrast, the polε-PVTE variant failed to produce full-length products even in the presence of PCNA. Nevertheless, PCNA clearly stimulated polε-PVTE activity (compare lanes −RFC and +RFC at 8 min, Fig. 4), although not sufficiently to restore synthesis to wild-type levels. The polε-KPFN variant, by contrast, produced full-length products after 8 min in the presence of PCNA, with an efficiency comparable to that of wild-type Pol ε. Thus, PCNA effectively rescues the activity of the polε-KPFN variant, likely by facilitating repeated dissociation and reassociation events during DNA synthesis.

### PCNA-dependent processivity

**Figure 5.**
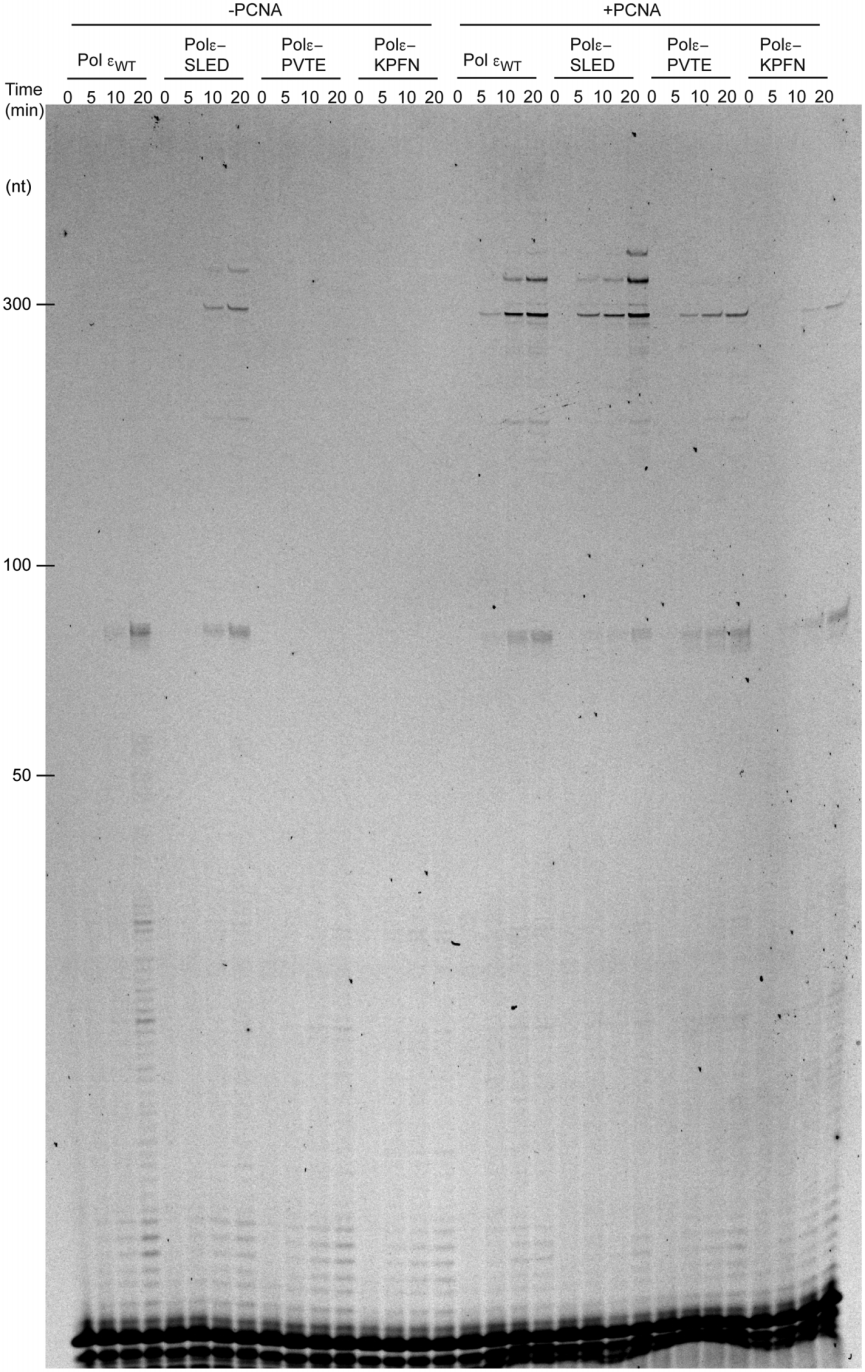
Denaturing PAGE (8%) showing the impact of absence/presence of PCNA on processive leading strand synthesis by wild type Pol ε and its variants. Extension of a 5′-TET-labeled 35–mer oligo annealed to M13mp18ssDNA (NEB). Reactions were performed at 40-fold molar excess of DNA substrate over polymerase to meet single-hit criteria. Individual reactions were stopped at 0, 2, 5, and 15 min, respectively.

Results from the primer-extension and holoenzyme assays indicated that mutations within the thumb domain affect polymerase processivity, although to differing extents among the variants. The effects of these mutations on processive DNA synthesis by Pol ε under single-hit conditions were, therefore, evaluated in the absence or presence of PCNA (Fig. 5).

In the absence of PCNA, the polε-SLED variant exhibited higher intrinsic processivity than wild-type Pol ε, whereas the polε-PVTE and polε-KPFN variants displayed reduced processivity relative to the wild-type enzyme (Fig. 5). These data indicate that the SLED mutation enhances the inherent processivity of Pol ε. Upon addition of PCNA, both wild-type Pol ε and the polε-SLED variant showed a marked increase in processivity (Fig. 5). Notably, the processivity of polε-SLED in the absence of PCNA was comparable to that of wild-type Pol ε in the presence of PCNA, further supporting the conclusion that this mutation confers an intrinsically more processive phenotype.

The polε-PVTE variant also exhibited increased processivity upon addition of PCNA. Similarly, the polε-KPFN variant showed a PCNA-dependent increase. However, the observed products were substantially weaker in intensity, indicating reduced overall DNA synthesis relative to the wild-type Pol ε and the other mutants. Given that polε-KPFN displayed near wild-type activity in the holoenzyme assay, one possible explanation for the reduced signal in the single-hit processivity assay is that this variant remains poorly processive even in the presence of PCNA. In the holoenzyme assay, the excess of enzyme likely permits repeated rebinding and extension of the DNA substrate, thereby compensating for the intrinsic processivity defect. Thus, the loss of processivity in polε-KPFN is masked under holoenzyme conditions but revealed under single-hit conditions.

### Impact of mutations in thumb domain on cellular fitness and spontaneous mutation rates *in vivo*

Because the polε-SLED, polε-PVTE, and polε-KPFN variants exhibited differences in both PCNA-independent and PCNA-dependent DNA synthesis *in vitro*, the *in vivo* effect of these mutations on cellular growth was examined. Heterozygous diploid strains in the E134 background carrying the pol2-SLED, pol2-PVTE, or pol2-KPFN alleles were constructed. Tetrad analysis revealed that haploid spores expressing pol2-SLED, pol2-PVTE, or pol2-KPFN displayed no detectable growth defect, as assessed by colony size in comparison to each other and to wild-type *POL2* strains (Supplementary Fig. S5A). Temperature sensitivity was evaluated by spot dilution assays, which showed that all variants grew equally well at 37°C and 30°C (Supplementary Fig. S5B). This result was expected, as none of the variants exhibited severe catalytic impairments relative to wild-type Pol ε *in vitro*, except under conditions involving difficult-to-replicate secondary structures in DNA.

Given the ability of the Pol ε-SLED variant to bypass DNA secondary structures and to exhibit increased processivity *in vitro*, we next examined whether the analyzed variants affect spontaneous mutation rates *in vivo*. Spontaneous mutation rates were quantified using fluctuation assays comprising two reversion assays and one forward mutation assay, as previously described [22]. As expected from the shift in balance toward proofreading associated with Pol ε-PVTE, the *pol2-PVTE* strain did not display a significant increase in mutation rate relative to the wild-type E134 strain. A very modest increase in mutation rate, with non-overlapping 95% confidence intervals, was observed at the *CAN1* locus, conferring resistance to canavanine, in the *pol2-KPFN* strain. In contrast, the *pol2-SLED* strain exhibited 1.5-fold and 3.5-fold increases in reversion rates at the *lys2::insEA14* and *his7-2* loci, respectively. In addition, a 1.9-fold increase in the forward mutation rate at the *CAN1* locus was observed (Table 1).

**Table 1.** Spontaneous mutation rates as observed in fluctuation assay

Strain	Mutation rate (x 10^−8^)*(95% confidence limits)					
	Lys^+^		His^+^		Can^r^	
	Absolute#	Relative(mut versus wt)	Absolute#	Relative(mut versus wt)	Absolute#	Relative(mut versus wt)
*POL2*	27.04(21.65–34.24)	1	1.54(1.19–2.23)	1	45.31(30.57–64.16)	1
*pol2-SLED*	40.8(36.13–58.53)	1.50	5.42(4.35–7.02)	3.5	84.12(75.28–106.79)	1.85
*pol2-PVTE*	27.50(26.37–33.83)	1.01	2.65(1.89–3.51)	1.72	71.75(55.24–78.01)	1.58
*pol2-KPFN*	31.32(24.75–40.81)	1.15	2.10(1.54–2.79)	1.36	73.93(66.41–80.77)	1.63

# Mutation rates are given as median of 20 independent cultures.

Taken together, these data demonstrate that the *pol2-SLED* mutation confers a modest mutator phenotype in the presence of functional mismatch repair. In contrast, mutations affecting regions of the thumb domain that are not unique to Pol ε, namely *pol2-PVTE* and *pol2-KPFN*, do not result in increased mutagenesis or lead to only a very minor increase, respectively. These findings suggest that the Pol ε-specific insertion within the thumb domain, the PTI, contributes to replication fidelity and that alterations in this region can subtly elevate spontaneous mutagenesis *in vivo*.

## Discussion

The acquisition of unique insertions within otherwise highly conserved protein folds is a recurring theme within the evolution of specialized functions [3, 4]. DNA polymerase ε (Pol ε) exemplifies this principle, as its catalytic core contains several Pol ε-specific insertions, including the P-domain, POPS, and a unique insertion within the thumb domain [5, 7, 8]. These elements have been implicated in features such as enhanced processivity, integration in the replisome, and replication fidelity. While the P-domain and POPS have been relatively well characterized, the functional contribution of the thumb domain insertion (PTI, residues 1116–1140 in yeast Pol2) has remained less well defined.

Recent work suggested that the PTI contributes to intrinsic processivity by promoting stable DNA binding by the thumb domain and that more extensive perturbations of this region reduce Pol ε processivity and compromise genome stability [10]. In parallel, cryo-EM structures of human Pol ε–PCNA complexes revealed that loops within or near the PTI region are positioned close to a PCNA protomer, raising the possibility that the PTI also participates in PCNA-mediated regulation of leading-strand synthesis [13, 16]. However, Pol ε already possesses multiple features that reduce its reliance on PCNA, including the P-domain and a stable association with the CMG helicase. This raises the question of whether the PTI evolved primarily to enhance processivity [10] or whether it instead serves a regulatory role in balancing processivity with fidelity.

To address this, alanine substitutions were made within the PTI (polε-SLED) and within two adjacent, structurally conserved thumb-domain loops (polε-PVTE and polε-KPFN). All three variants supported normal cellular growth and showed no detectable temperature sensitivity, indicating that these mutations do not compromise essential Pol ε functions *in vivo*. Nonetheless, biochemical analyses revealed pronounced and mechanistically informative differences among the variants.

Substitutions in the conserved thumb-domain loops (polε-PVTE and polε-KPFN) impaired DNA synthesis on templates containing secondary structures. These variants stalled at a hairpin element, and polε-PVTE in particular displayed enhanced primer degradation, consistent with a shift toward exonucleolytic proofreading. Given the established role of the thumb domain in stabilizing the contact with the nascent DNA duplex, these phenotypes are most readily explained by altered DNA–enzyme interactions. Destabilization of the primer-template interaction likely increases the residence time of a mismatched or stalled intermediate, thereby favoring transfer of the primer terminus to the exonuclease site. These effects were only partially alleviated by PCNA, indicating that PCNA cannot fully compensate for defects arising from perturbation of conserved thumb-domain contacts with DNA.

In contrast, substitutions within the PTI produced a markedly different phenotype. The polε-SLED variant bypassed secondary structures more efficiently than wild-type Pol ε, generated fewer degradation products, and exhibited substantially increased intrinsic processivity, even in the absence of PCNA. Notably, this enhanced processivity correlated with a modest but reproducible increase in spontaneous mutation rates *in vivo*. Thus, altering the PTI shifts Pol ε toward a more processive but less accurate mode of DNA synthesis, in contrast to the previously reported reduced processivity [10].

Importantly, none of the thumb-domain mutations abolished PCNA responsiveness. Both polε-SLED and polε-KPFN displayed PCNA-dependent increases in processivity, indicating that the thumb domain is not essential for PCNA engagement. Instead, these findings suggest that the PTI primarily modulates intrinsic polymerase behavior rather than serving as a dominant PCNA-interaction module. The observation that polε-SLED achieves processivity levels comparable to PCNA-stimulated wild-type Pol ε further supports a hypothesis that the PTI normally acts to restrain intrinsic processivity.

Collectively, our data support a model in which the PTI evolved to fine-tune the balance between processivity and fidelity during leading-strand synthesis. Excessive processivity, while advantageous for uninterrupted DNA synthesis, may limit opportunities for error correction by reducing polymerase dissociation and proofreading engagement. In the context of continuous leading-strand replication, even modest increases in error rates could have deleterious consequences. By tempering intrinsic processivity, the PTI appears to promote a replication mode that preserves high fidelity while remaining sufficiently processive for efficient genome duplication.

Together, our findings reveal the PTI as a critical regulatory element that enables Pol ε to achieve an optimal balance between processivity and accuracy, underscoring how subtle structural modifications within conserved enzymes can have profound functional consequences.

## Supplementary Material

gkag282_Supplemental_File

## Data Availability

The data underlying this article will be shared on request to the corresponding author.

## References

[1] Kohlstaedt LA, Wang J, Friedman JM et al. Crystal structure at 3.5 Å resolution of HIV-1 reverse transcriptase complexed with an inhibitor. Science. 1992;256:1783–90. 10.1126/science.1377403. 1377403

[2] Johansson E, Macneill SA. The eukaryotic replicative DNA polymerases take shape. Trends Biochem Sci. 2010;35:339–47. 10.1016/j.tibs.2010.01.004. 20163964

[3] Todd AE, Orengo CA, Thornton JM. Evolution of function in protein superfamilies, from a structural perspective. J Mol Biol. 2001;307:1113–43. 10.1006/jmbi.2001.4513. 11286560

[4] Orengo CA, Thornton JM. Protein families and their evolution—a structural perspective. Annu Rev Biochem. 2005;74:867–900. 10.1146/annurev.biochem.74.082803.133029. 15954844

[5] Hogg M, Osterman P, Bylund GO et al. Structural basis for processive DNA synthesis by yeast DNA polymerase varepsilon. Nat Struct Mol Biol. 2014;21:49–55. 10.1038/nsmb.2712. 24292646

[6] Asturias FJ, Cheung IK, Sabouri N et al. Structure of *Saccharomyces cerevisiae* DNA polymerase epsilon by cryo-electron microscopy. Nat Struct Mol Biol. 2006;13:35–43. 10.1038/nsmb1040. 16369485

[7] Shcherbakova PV, Pavlov YI, Chilkova O et al. Unique error signature of the four-subunit yeast DNA polymerase epsilon. J Biol Chem. 2003;278:43770–80. 10.1074/jbc.M306893200. 12882968

[8] Meng X, Wei L, Devbhandari S et al. DNA polymerase epsilon relies on a unique domain for efficient replisome assembly and strand synthesis. Nat Commun. 2020;11:2437. 10.1038/s41467-020-16095-x. 32415104 PMC7228970

[9] Ter Beek J, Parkash V, Bylund GO et al. Structural evidence for an essential Fe-S cluster in the catalytic core domain of DNA polymerase. Nucleic Acids Res. 2019;47:5712–22. 10.1093/nar/gkz248. 30968138 PMC6582351

[10] Ahmad S, Zhang S, Meng X. A conserved thumb domain insertion in DNA polymerase epsilon supports processive DNA synthesis. Nucleic Acids Res. 2025;53:gkaf190. 10.1093/nar/gkaf190. 40105244 PMC11920795

[11] Steitz TA . DNA polymerases: structural diversity and common mechanisms. J Biol Chem. 1999;274:17395–8. 10.1074/jbc.274.25.17395. 10364165

[12] Ganai RA, Bylund GO, Johansson E. Switching between polymerase and exonuclease sites in DNA polymerase epsilon. Nucleic Acids Res. 2015;43:932–42. 10.1093/nar/gku1353. 25550436 PMC4333401

[13] Roske JJ, Yeeles JTP. Structural basis for processive daughter-strand synthesis and proofreading by the human leading-strand DNA polymerase Pol epsilon. Nat Struct Mol Biol. 2024;31:1921–31. 10.1038/s41594-024-01370-y. 39112807 PMC11638069

[14] Franklin MC, Wang J, Steitz TA. Structure of the replicating complex of a pol alpha family DNA polymerase. Cell. 2001;105:657–67. 10.1016/S0092-8674(01)00367-1. 11389835

[15] Perez-Arnaiz P, Lazaro JM, Salas M et al. Involvement of phi29 DNA polymerase thumb subdomain in the proper coordination of synthesis and degradation during DNA replication. Nucleic Acids Res. 2006;34:3107–15. 10.1093/nar/gkl402. 16757576 PMC1475753

[16] He Q, Wang F, Yao NY et al. Structures of the human leading strand Polε–PCNA holoenzyme. Nat Commun. 2024;15:7847. 10.1038/s41467-024-52257-x. 39245668 PMC11381554

[17] Henricksen LA, Umbricht CB, Wold MS. Recombinant replication protein A: expression, complex formation, and functional characterization. J Biol Chem. 1994;269:11121–32. 10.1016/S0021-9258(19)78100-9. 8157639

[18] Gomes XV, Gary SL, Burgers PM. Overproduction in *Escherichia coli* and characterization of yeast replication factor C lacking the ligase homology domain. J Biol Chem. 2000;275:14541–9. 10.1074/jbc.275.19.14541. 10799539

[19] Ayyagari R, Impellizzeri KJ, Yoder BL et al. A mutational analysis of the yeast proliferating cell nuclear antigen indicates distinct roles in DNA replication and DNA repair. Mol Cell Biol. 1995;15:4420–9. 10.1128/MCB.15.8.4420. 7623835 PMC230682

[20] Isoz I, Persson U, Volkov K et al. The C-terminus of Dpb2 is required for interaction with Pol2 and for cell viability. Nucleic Acids Res. 2012;40:11545–53. 10.1093/nar/gks880. 23034803 PMC3526264

[21] Shcherbakova PV, Kunkel TA. Mutator phenotypes conferred by MLH1 overexpression and by heterozygosity for mlh1 mutations. Mol Cell Biol. 1999;19:3177–83. 10.1128/MCB.19.4.3177. 10082584 PMC84111

[22] Aksenova A, Volkov K, Maceluch J et al. Mismatch repair-independent increase in spontaneous mutagenesis in yeast lacking non-essential subunits of DNA polymerase epsilon. PLoS Genet. 2010;6:e1001209. 10.1371/journal.pgen.1001209. 21124948 PMC2987839

