# Supplementary material for: A thumb-domain insertion balances processivity and fidelity in DNA polymerase ε
Source: Nucleic Acids Res. 2026 Mar 31;54(6):gkag282. doi: 10.1093/nar/gkag282 (PMC13036494; doi:10.1093/nar/gkag282)
Supplement: gkag282_Supplemental_File [file gkag282_supplemental_file.pdf]

A thumb-domain insertion balances processivity and fidelity in DNA polymerase ε

Noopur Singh[1] , Göran O. Bylund[1] and Erik Johansson[1]

1) Department of Medical Biochemistry and Biophysics, Umeå University, 901 87 Umeå, Sweden

Corresponding author: Erik Johansson, e-mail: erik.tm.johansson@umu.se

Table of contents:

Supplementary Table 1……………………………………………………………….3

Supplementary figure 1…………………………………………………………………4

Supplementary figure 2…………………………………………………………………5

Supplementary figure 3…………………………………………………………………6

Supplementary figure 4…………………………………………………………………7

Supplementary figure 5…………………………………………………………………8

Supplementary Table 1

Table S1. Oligonucleotide sequences used for generation and screening of thumb-domain mutants.

Primers for cloning	Sequence (5’-3’)
SLED_1	TTAGATCCA**GCTGCGGCAGCT**CTGGATATCAGAACC
SLED_2	GGTTCTGATATCCAG**AGCTGCCGCAGC**TGGATCTAATGTCC
KPFN_1	TATATTATTAGTTCA**GCAGCTGCCGCT**GCACCTGTTACTGAACG
KPFN_2	CAGTAACAGGTGC**AGCGGCAGCTGC**TGAACTAATAATATATTTAC
PVTE_1	CCTTTCAATGCA**GCTGCTGCTGCA**CGAGCCATTCCAG
PVTE_2	AATGGCTCG**TGCAGCAGCAGC**TGCATTGAAAGGTTTTG
Primers for screening	Sequence 5’-3’
Screen-SLED-R	TTCTGATATCCAGAGCTGCCGC
Screen-PVTE-R	GCGACTGGAATGGCTCGTGCAGCAG
Screen-KPFN-R	CAGTAACAGGTGCAGCGGCAGCTGC
Pol2-25	ATGAGACGCACAGTGAAAACAC

Supplementary Figure1

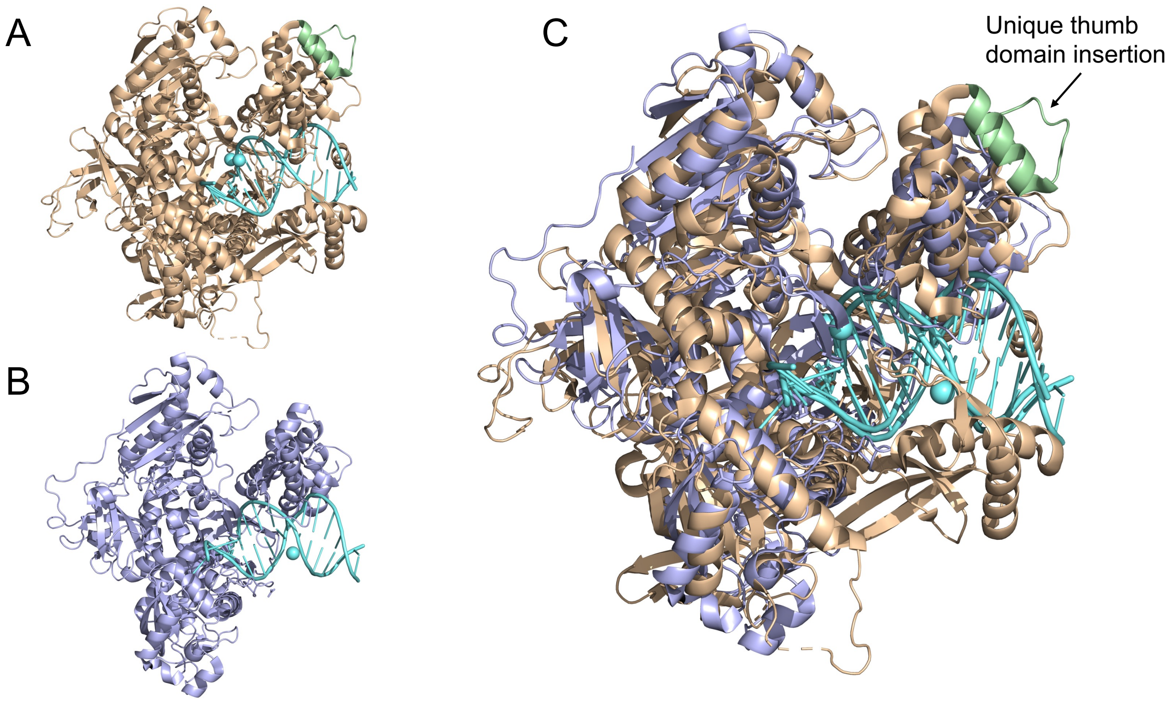

Fig S1. Catalytic core region of (A) Polε (PDB ID 4m8o(1)) and (B) Polδ (PDB ID 3iay(2)) (C) Structural alignment of catalytic fragments of Polε (wheat) and Polδ (light blue). Unique thumb domain insertion (residue 1116-1140 in yeast) is shown in pale green color.

Supplementary Figure 2

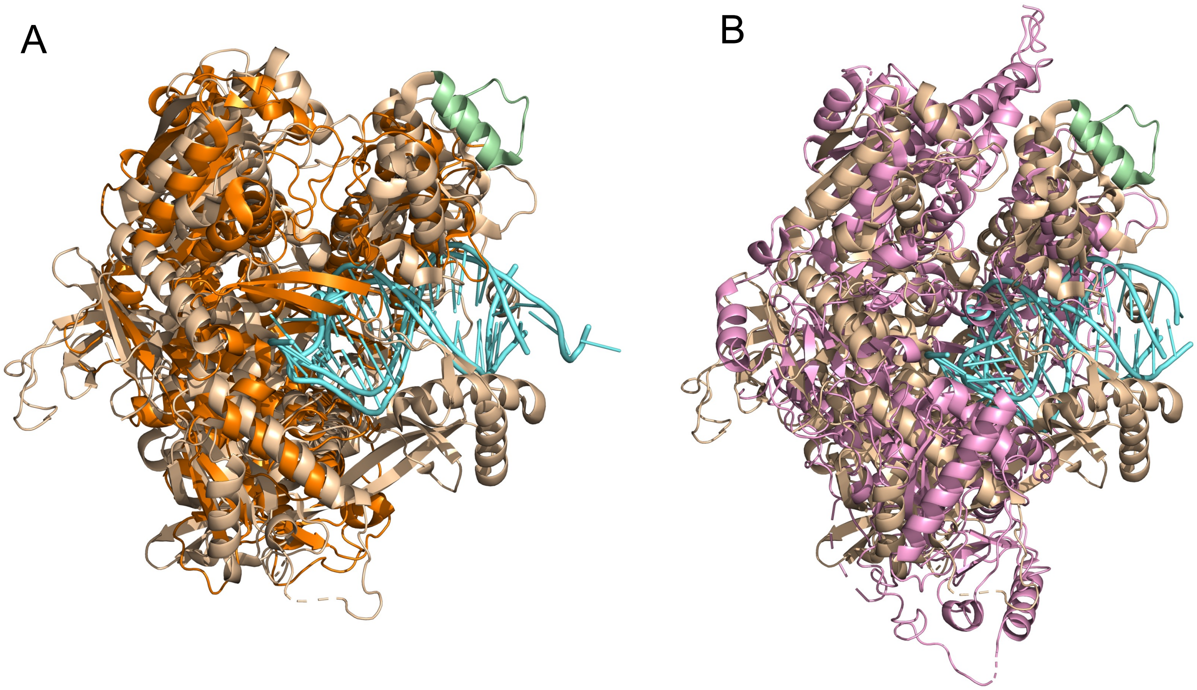

Fig S2. Structural alignment of catalytic core fragment of yeast Polε (PDB ID 4m8o(1)) shown in wheat color with (A) RB69 DNA polymerase (PDB ID 3nci(3)) and (B) Polζ (PDB ID 6v93(4)) Unique thumb domain insertion in Polε is highlighted in pale green color.

Supplementary Figure 3

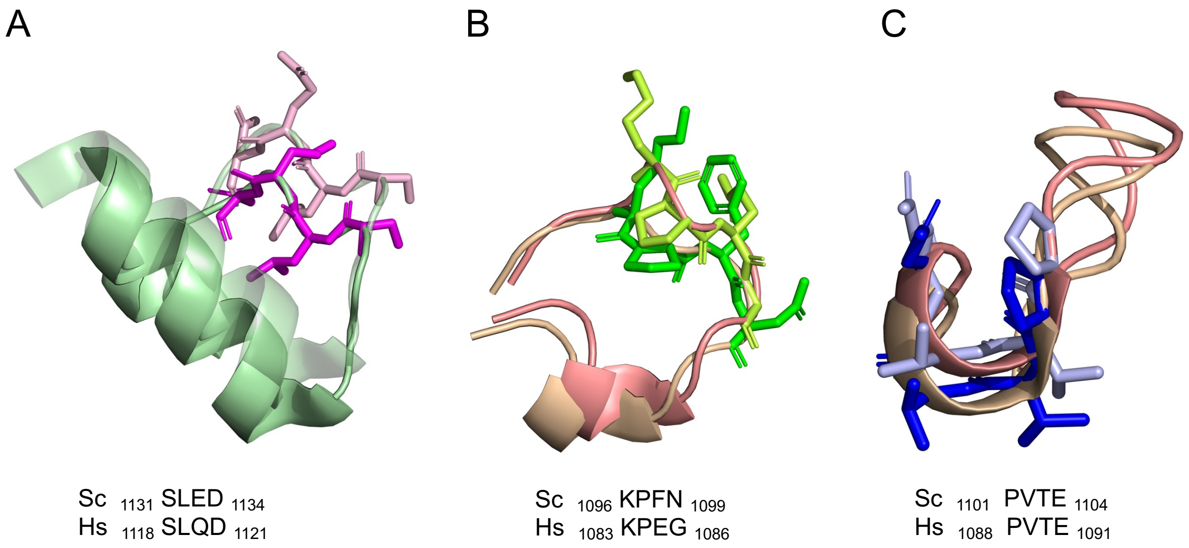

Fig S3. Structural superpositions between yeast and human Pol ε showing the residues selected for mutation as sticks. Corresponding residues in human are shown in lighter shades of the same color. *Sc- Saccharomyces cerevisiae* (PDB ID 4m8o(1)), *Hs-Homo sapiens* (PDB ID 9f6f(5)).

Supplementary Figure 4

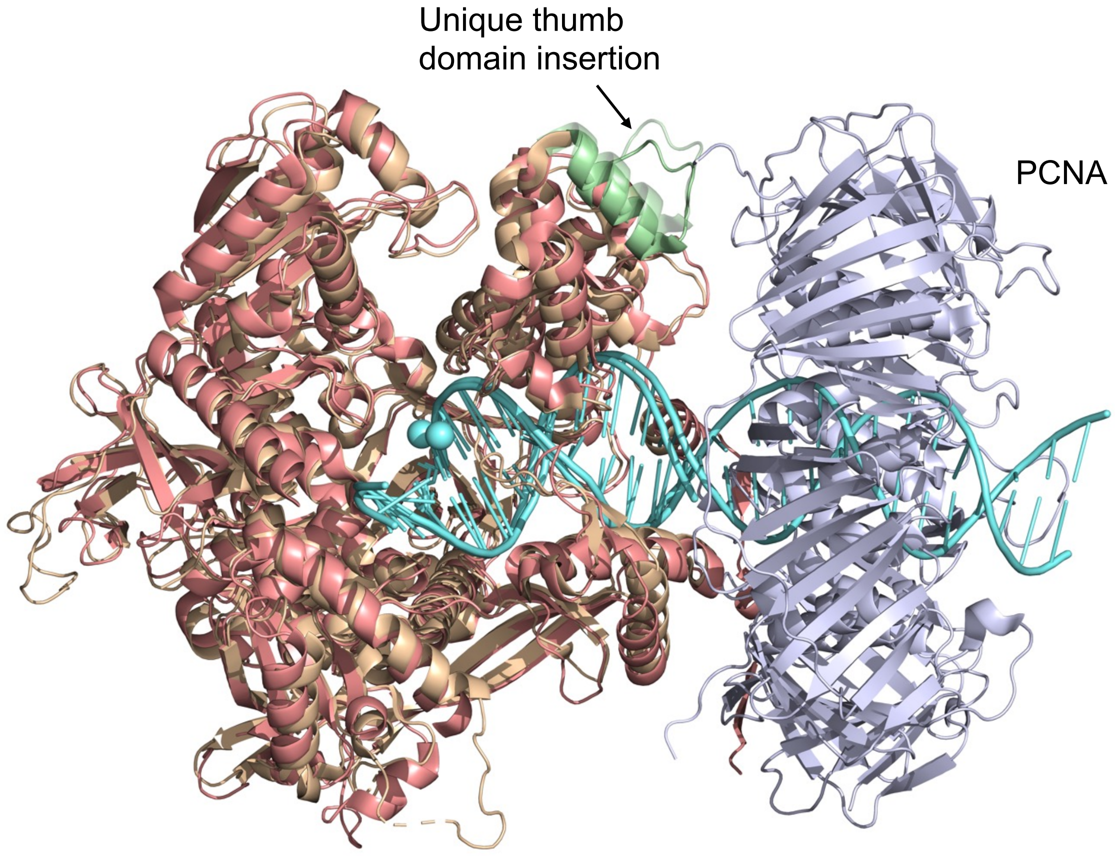

Fig S4. Structural alignment of catalytic core fragment of yeast Polε (PDB ID 4m8o(1)) shown in wheat color with human Polε-PCNA complex (PDB ID 9f6f(5)) shown in salmon color. Unique thumb domain insertion is shown in pale green color (transparent for PDB ID 9f6f(5)).

Supplementary Figure 5

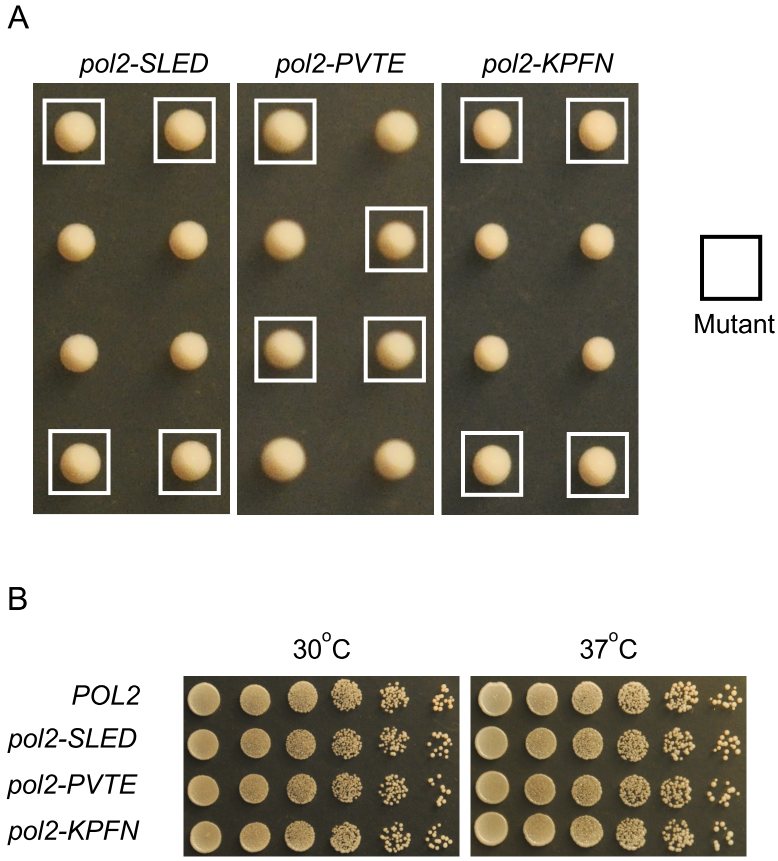

Fig S5. In vivo analysis showing the impact of thumb domain mutants of Pol ε on cell fitness. (A) Tetrad analysis of diploid yeast E134 strains heterozygous for *POL2/pol2-SLED, POL2/pol2-PVTE, POL2/pol2-KPFN*. (B) Spot dilution assay to compare temperature sensitivity of Polε variants. Overnight cultures were diluted to OD_{600}=1 and followed by a 5-fold serial dilution. 5 µl from each dilution was plated on YPD agar plates. Plates were incubated for 2 days at 30°C and 30°C, respectively.

References

1. Hogg, M., Osterman, P., Bylund, G.O., Ganai, R.A., Lundstrom, E.B., Sauer-Eriksson, A.E. and Johansson, E. (2014) Structural basis for processive DNA synthesis by yeast DNA polymerase varepsilon. *Nat Struct Mol Biol*, **21**, 49-55.
2. Swan, M.K., Johnson, R.E., Prakash, L., Prakash, S. and Aggarwal, A.K. (2009) Structural basis of high-fidelity DNA synthesis by yeast DNA polymerase delta. *Nature structural & molecular biology*, **16**, 979-986.
3. Wang, M., Xia, S., Blaha, G., Steitz, T.A., Konigsberg, W.H. and Wang, J. (2011) Insights into base selectivity from the 1.8 A resolution structure of an RB69 DNA polymerase ternary complex. *Biochemistry*, **50**, 581-590.
4. Malik, R., Johnson, R.E., Prakash, L., Prakash, S., Ubarretxena-Belandia, I. and Aggarwal, A.K. (2022) Cryo-EM structure of translesion DNA synthesis polymerase zeta with a base pair mismatch. *Nat Commun*, **13**, 1050.
5. Roske, J.J. and Yeeles, J.T.P. (2024) Structural basis for processive daughter-strand synthesis and proofreading by the human leading-strand DNA polymerase Pol epsilon. *Nat Struct Mol Biol*.
